# Supplementary material for: IoT-based control and monitoring system for hydroponic plant growth using image processing and mobile applications
Source: PeerJ Comput Sci. 2025 Mar 28;11:e2763. doi: 10.7717/peerj-cs.2763 (PMC12190697; doi:10.7717/peerj-cs.2763)
Supplement: Supplemental Information 5 [file peerj-cs-11-2763-s005.pdf]

HydroFarm Apps SUS Test

Kenali nutrisi dan kondisi pada tanaman hidroponik mu!

HydroFarm merupakan sistem pengendalian dan pengawan tumbuh kembang tanaman hidroponik berbasis Internet of Things, Image Processing, dan Mobile Apps

Aplikasinya:
https://drive.google.com/drive/folders/1TmvZqtswlavcoe187fB3oSA-_0D7t0nR?usp=sharing

* Menunjukkan pertanyaan yang wajib diisi

1. Email *

____

2. Nama *

____

3. Saya merasa nyaman menggunakan HydroFarm *

Tandai satu oval saja.

	1	2	3	4	5	
San	◯	◯	◯	◯	◯	Sangat Setuju

4. Saya merasa fitur-fitur dalam HydroFarm terlalu rumit untuk digunakan *

Tandai satu oval saja.

	1	2	3	4	5	
Sang	◯	◯	◯	◯	◯	Sangat Setuju

5. Saya merasa bahwa HydroFarm mudah digunakan *

Tandai satu oval saja.

	1	2	3	4	5	
Sang	◯	◯	◯	◯	◯	Sangat Setuju

6. Saya pikir saya akan memerlukan bantuan teknis untuk dapat menggunakan HydroFarm dengan baik *

Tandai satu oval saja.

	1	2	3	4	5	
Sang	◯	◯	◯	◯	◯	Sangat Setuju

7. Fitur-fitur yang ada dalam HydroFarm terintegrasi dengan baik *

Tandai satu oval saja.

	1	2	3	4	5	
Sang	◯	◯	◯	◯	◯	Sangat Setuju

8. Saya merasa ada terlalu banyak inkonsistensi dalam HydroFarm *

Tandai satu oval saja.

	1	2	3	4	5	
San(	◯	◯	◯	◯	◯	Sangat Setuju

9. Saya merasa kebanyakan orang dapat mempelajari cara menggunakan HydroFarm dengan cepat *

Tandai satu oval saja.

	1	2	3	4	5	
San(	◯	◯	◯	◯	◯	Sangat Setuju

10. Saya merasa HydroFarm terlalu membingungkan untuk digunakan. *

Tandai satu oval saja.

	1	2	3	4	5	
San(	◯	◯	◯	◯	◯	Sangat Setuju

11. Saya merasa percaya diri ketika menggunakan HydroFarm. *

Tandai satu oval saja.

	1	2	3	4	5	
San(	◯	◯	◯	◯	◯	Sangat Setuju

12. Saya perlu mempelajari banyak hal sebelum saya bisa menggunakan HydroFarm. *

Tandai satu oval saja.

	1	2	3	4	5	
San[illegible]	◯	◯	◯	◯	◯	Sangat Setuju

13. Saran dan Masukan *

____

____

____

____

____

Konten ini tidak dibuat atau didukung oleh Google.

Google Formulir
