# Supplementary material for: IoT-based control and monitoring system for hydroponic plant growth using image processing and mobile applications
Source: PeerJ Comput Sci. 2025 Mar 28;11:e2763. doi: 10.7717/peerj-cs.2763 (PMC12190697; doi:10.7717/peerj-cs.2763)
Supplement: Supplemental Information 6 [file peerj-cs-11-2763-s006.pdf]

HydroFarm Apps SUS Test

Know the Nutrition and Condition of Your Hydroponic Plants!

HydroFarm is a system for controlling and monitoring the growth of hydroponic plants, based on the Internet of Things, Image Processing, and Mobile Apps.

The App:
https://drive.google.com/drive/folders/1TmvZqtswlavcoe187fB3oSA-_0D7t0nR?usp=sharing

* Menunjukkan pertanyaan yang wajib diisi

1. Email *

2. Name *

3. I feel comfortable using HydroFarm *

Tandai satu oval saja.

	1	2	3	4	5	
Stro	◯	◯	◯	◯	◯	Strongly agree

4. I find the features in HydroFarm too complex to use *

Tandai satu oval saja.

	1	2	3	4	5	
Stro	◯	◯	◯	◯	◯	Strongly agree

5. I find HydroFarm easy to use *

Tandai satu oval saja.

	1	2	3	4	5	
Stro	◯	◯	◯	◯	◯	Strongly agree

6. I think I would need technical support to use HydroFarm effectively *

Tandai satu oval saja.

	1	2	3	4	5	
Stro	◯	◯	◯	◯	◯	Strongly agree

7. The features in HydroFarm are well-integrated *

Tandai satu oval saja.

	1	2	3	4	5	
Stro	◯	◯	◯	◯	◯	Strongly agree

8. I find too much inconsistency within HydroFarm *

Tandai satu oval saja.

	1	2	3	4	5	
Stro	◯	◯	◯	◯	◯	Strongly agree

9. I believe most people can learn to use HydroFarm quickly *

Tandai satu oval saja.

	1	2	3	4	5	
Stro	◯	◯	◯	◯	◯	Strongly agree

10. I find HydroFarm too confusing to use *

Tandai satu oval saja.

	1	2	3	4	5	
Stro	◯	◯	◯	◯	◯	Strongly agree

11. I feel confident using HydroFarm *

Tandai satu oval saja.

	1	2	3	4	5	
Stro	◯	◯	◯	◯	◯	Strongly agree

12. I need to learn a lot before I can effectively use HydroFarm *

Tandai satu oval saja.

	1	2	3	4	5	
Stro	◯	◯	◯	◯	◯	Strongly agree

13. Suggestions *

____
____
____
____
____

Konten ini tidak dibuat atau didukung oleh Google.

Google Formulir
